# HIV and Messenger RNA (mRNA) Vaccine

**DOI:** 10.7759/cureus.16197

**Published:** 2021-07-05

**Authors:** Khizer Khalid, Jaskamal Padda, Anwar Khedr, Dina Ismail, Ujala Zubair, Ola A Al-Ewaidat, Sandeep Padda, Ayden Charlene Cooper, Gutteridge Jean-Charles

**Affiliations:** 1 Internal Medicine, Gutteridge Jean-Charles (JC) Medical Center, Orlando, USA; 2 Family Medicine, Gutteridge Jean-Charles (JC) Medical Center, Orlando, USA; 3 Internal Medicine, Advent Health & Orlando Health Hospital/Gutteridge Jean-Charles (JC) Medical Center, Orlando, USA

**Keywords:** hiv, vaccine, lipid nanoparticles, pandemic, mrna

## Abstract

Human immunodeficiency virus (HIV) is a part of the lentivirus genus of the retroviridae family that incorporates its genome into the host DNA via a series of complex steps. HIV can be classified into two types, HIV-type 1 (HIV-1) and HIV-type 2 (HIV-2), with HIV-1 being the most common type worldwide. Seventy-six million people have been infected since the start of the pandemic, with a mortality rate of 33 million. Even after 40 years, no cure has been developed for this pandemic. The development of the mRNA vaccine has led to further research for the utilization of mRNA vaccine in HIV, in attempts to create a prophylactic and therapeutic treatment. Although messenger RNA (mRNA) vaccine has been around for many years, it has recently drawn attention due to its role and response in the unforeseen coronavirus pandemic. mRNA vaccine has faced its fair-share of challenges, but it also offers many advantages compared to conventional vaccines such as safety, efficacy, rapid preparation, and versatility. mRNA vaccine has shown promising results and has great potential. In this review, we discuss the types of mRNA vaccine, along with development, delivery, advantages, challenges, and how we are working to overcome these challenges.

## Introduction and background

Since the start of the human immunodeficiency virus/acquired immunodeficiency syndrome (HIV/AIDS) pandemic, 76 million people have been infected with 33 million deaths. In 2019, there was a worldwide prevalence of 38 million people. Even after four decades, no cure has been developed for the pandemic [1]. The current available antiretroviral drugs are being used to treat and prevent HIV which has helped control the disease progression and prevent further complications for many patients. However, there are many drawbacks of these medications including their side effects, cost, and the need of strict adherence to a medication schedule to prevent drug resistance [2]. This necessitates the need for a therapeutic or prophylactic vaccine which is safe and effective.

Over the past three decades, the development of an effective HIV vaccine has been extremely difficult. Only one of the available HIV vaccine efficacy trials showed modest efficiency, with an efficacy of 31% in the RV144 trial in Thailand [3]. There has been a tremendous effort to develop more effective vaccines using nucleic acid (DNA/RNA) technology. Although messenger RNA (mRNA) vaccines showed promising results as an alternative to the conventional methods, their use has been restricted due to high intrinsic immunogenicity, easy degradation, and inefficacious in vivo delivery [4]. During the severe acute respiratory syndrome coronavirus 2 (SARS-CoV-2) pandemic, there have been successful innovations to use lipid nanoparticles (LNPs) as a delivery and adjuvant system for mRNA vaccines to overcome these drawbacks. This gives hope to develop an effective HIV vaccine using mRNA-LNP technology [5]. 

Here, we will review the mechanism of HIV infection, and the history, types, advantages as well as challenges of developing HIV mRNA vaccine. 

## Review

What is HIV

HIV belongs to the retroviridae family's Lentivirus genus. It is classified into two kinds: HIV-type 1 (HIV-1) and HIV-type 2 (HIV-2) [6]. HIV-1 is the most common type globally [7]. HIV-2 is the predominant type in western and central Africa but has reached Europe and India. Both viruses potentially lead to the development of AIDS, depending on the characteristics of the infecting viral particles and the host immune response. The retrovirus genome consists of two indistinguishable RNA strands. It is characterized by the presence of certain genes that encode specific proteins performing different functions to promote the tropism of the virus, as illustrated in Table 1 [6].

**Table 1:** HIV-1 gene-encoded proteins and their functions. Gag: group antigen; Env: envelope; Tat: trans-activator of transcription; Vif: virion infectivity factor; Vpu: viral protein U; Rev: regulator of expression of virion proteins; Nef: negative factor; gp: glycoprotein.

Gene	Function
Gag	Encodes p24, p7, and p6 core proteins and p17 matrix protein
Pol	Encodes for reverse transcriptase, integrase, and protease; Reverse transcriptase transforms viral RNA into DNA, integrase incorporates viral DNA into the chromosomal DNA of the host, and protease cleaves huge gag and Pol protein precursors into their components, all of which are required for viral replication.
Env	Encodes for gp120 and gp41, the glycoproteins of the viral envelope which target the receptors of the cell surface
Tat	Encodes for the Tat protein, which is produced early after infection and increases HIV gene expression
Vif	Encodes for a small protein called Vif that promotes the infectivity of the viral particles
Vpu	Encodes for a protein called Vpu that takes part in the arrest of the cell cycle
Rev	Encodes for a protein called Rev that regulates the nuclear export of the mRNA
Nef	Encodes for the Nef protein, which modulates cellular signalling and increases the downregulation of the cell surface’s CD4 receptors, allowing viral replication.

Mechanisms of infection and pathogenesis

**Figure 1:**
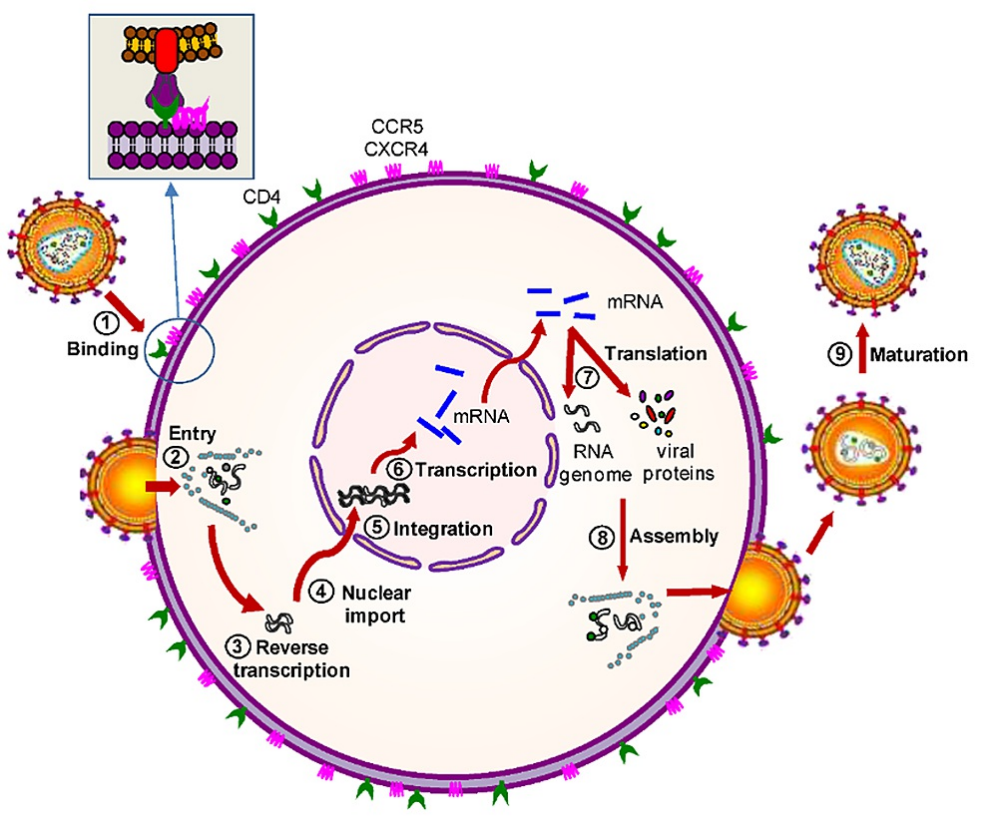
Steps of HIV-1 replication cycle. Copyright/License Licensee MDPI, Basel, Switzerland. This figure is from an open access article distributed under the terms and conditions of the Creative Commons Attribution (CC BY) license. (http://creativecommons.org/licenses/by/4.0/) No modifications were made to the original figure. Herrera-Carrillo E, Berkhout B: Bone marrow gene therapy for HIV/AIDS. Viruses. 2015, 7:3910–3936. 10.3390/v7072804 [10].

Sexual contact, exposure to infected blood, and perinatal transmission are the modes of transmission of HIV [7]. It is estimated that sexual exposure is responsible for more than 90% of HIV infections in the United States [8]. During sexual transmission, HIV first targets CCR5 expressing CD4+ T-cells in the endocervix and the rectum [9]. It also targets CXCR4 receptors on macrophages, T-cells, and dendritic cells (DC). The HIV-1 then follows its replication cycle in cells, as illustrated in (Figure 1) [10]. Population bottleneck occurs in HIV transmission in acute infections, where one or a few genetic variants of the HIV generates a productive infection [11]. Then, the HIV reservoir is set up very early in the course of the infection. HIV then travels from the infecting site to secondary lymphoid organs and tissues within one week of exposure and can be detected in most tissues and blood within two weeks [9]. The majority of CCR5-expressing CD4 T cells get depleted within the blood, lymph nodes, and gastrointestinal tract, which is the most affected by this depletion [12].

What is mRNA vaccine and how does it work?

From the mRNA World to the Clinic

**Figure 2:**
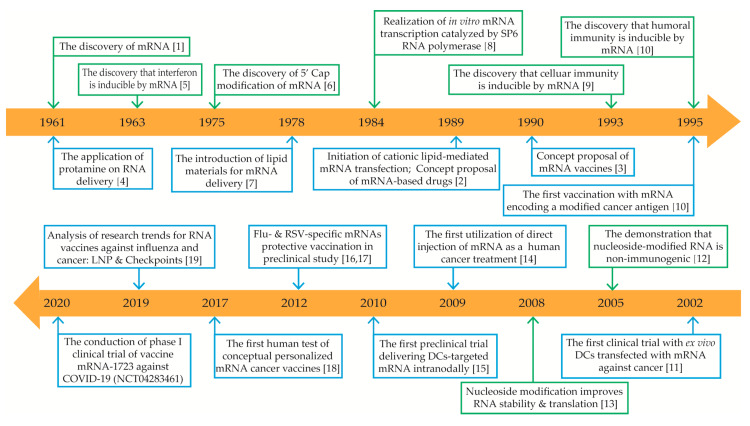
mRNA-based drugs major breakthroughs timeline. Green boxes represent discoveries and advances in mRNA mechanisms; blue boxes represent discoveries and advances in mRNA-based drug applications. mRNA: messenger RNA; 5′ cap: five-prime cap; LNP: lipid nanoparticles; COVID-19: coronavirus disease 2019; DCs: dendritic cells. Copyright/License Licensee MDPI, Basel, Switzerland. This figure is from an open access article distributed under the terms and conditions of the Creative Commons Attribution (CC BY) license. (http://creativecommons.org/licenses/by/4.0/) No modifications were made to the original figure. Xu S, Yang K, Li R, Zhang L: mRNA vaccine era-mechanisms, drug platform and clinical prospection. Int J Mol Sci. 2020, 21:6582. 10.3390/ijms21186582 [21].

The molecule known as mRNA has been the unexpected star of the coronavirus pandemic response. The technology employed in Moderna and Pfizer vaccines is based on its premise [13]. However, mRNA is not a brand-new invention from the lab, it is found in every cell of an organism and has been around for billions of years. Scientists believe that RNA existed before DNA in the earliest biological forms [14]. mRNA serves as a bridge between the translation of protein-coding DNA and the cytoplasmic synthesis of proteins by ribosomes [4]. Brenner et al. were the first to discover the molecule in 1961 [15]. However, it was not until 1989 that the concept of the mRNA-based drug was established, when Malone et al. revealed that while wrapped in a cationic lipid package, mRNA could be transfected and expressed in a variety of eukaryotic cells [16]. This was successfully demonstrated a year later, when intramuscular injection of mRNA in mice resulted in the expression of the desired protein, thus revealing the prospect of creating an mRNA vaccine [17]. Since then, advances in mRNA structural studies have been rapidly expanding. Nevertheless, before the twenty-first century, the idea of an mRNA vaccine was much less intriguing. The reason behind it is divided into three components. It has been found that mRNAs are not as stable as DNA or proteins and are susceptible to degradation by RNase or ribonuclease. On the other hand, they elicit a robust immunological response, just like a viral invasion. As a result, mRNA vaccinations pose significant safety concerns. The last reason would be that the delivery efficiency of mRNA vaccine into a living organism has a high failure rate [18]. These concerns have majorly resulted in a lack of significant investment in the development of mRNA vaccines. Fortunately, major technological innovations in RNA chemistry stabilization, biology, and systems of delivery into the cell have made it possible to overcome these challenges [19,20]. mRNA has now become a promising platform in vaccine development for the prevention, control, and treatment of infection and cancer (Figure 2) [21].

Mechanism

The concept for developing an mRNA vaccine is rather simple. The first step is to identify the antigen of choice from the target pathogen. The selected gene will then be sequenced, synthesized, and cloned to create a DNA template plasmid (pDNA). In vitro, this template will be transcribed onto an mRNA vaccine that can be delivered into the subject. In vivo, the mRNA vaccine will mimic a viral infection by using the host cell to translate mRNA into the appropriate antigen, triggering powerful humoral and cellular immune responses. The transmembrane domain and signal peptide dictate the antigen's subsequent cellular position. This could be integrated into the original nucleotide sequences in order to send the targeted antigen to the specific cellular compartment [20]. As a result, the antigen can either be expressed as cytoplasmic, secreted, or membrane-bound. Since it is entirely synthetic, nearly any sequence could be designed, synthesized, and delivered as an mRNA vaccine to be tested in vivo within a short amount of time. In practice, antigen sequences can be tagged with targeting sequences for major histocompatibility complex (MHC) class II compartments, MHC class I trafficking signals, or immunodominant helper CD4 T cell epitopes. Thus, improving antigen presentation efficiency and cellular immune responses [20]. The host innate immune system’s capacity to sense and respond to RNA sequences of viral origin has made it plausible for mRNA vaccines to produce an efficient innate response, along with the generation of chemokines and cytokines such as interleukin-12 (IL-12) and tumor necrosis factor (TNF) at the injection site, which is critical in the production of successful adaptive immune responses against the encoded antigen [22-25].

mRNA Vaccine Production and Purification

All kinds of mRNA vaccines share the same structural elements: a cap structure, 5’UTR, 3’UTR, open reading frame (OFR), and a poly(A) tail (40-120 adenosine residues). They are produced in a cell-free environment by enzymatic transcription of a pDNA template. It is only necessary to substitute the sequence encoding for the target antigen without affecting the overall physio-chemical structure of the mRNA vaccine [25,26]. 

The first step in mRNA vaccine production is to produce a template pDNA containing a promoter sequence with a high binding affinity to a DNA-dependent RNA polymerase. Next, the circular plasmid DNA is cut with a restriction enzyme and turned into a single-stranded linear molecule. This template is then transcribed in vitro (IVT) using a DNA-dependent RNA polymerase enzyme that advances through the template until it reaches the end. A DNase degrades the template while a cap [m7Gp3N] is enzymatically added to the 5’ end of the mRNA [27]. This cap can be synthetically replaced with an analog that conveys more advantages to the mRNA sequence. Its presence is required for an effective translation in vivo and serves to protect mRNA from nuclease digestion within the cell [28]. After synthesis, mRNA is purified to remove reaction components such as enzymes, leftover template DNA, shortened transcripts, and abnormal double-stranded transcripts. Because impurities might activate non-specific or unwanted innate immune responses, a highly purified RNA material is crucial for the effectiveness of an mRNA vaccination [29]. Following purification, mRNA is either stored in a final buffer or mixed with the delivery system for use. This is how practically any mRNA sequence can be fabricated, with the advantages of low batch-to-batch variability and time and money-saving over existing vaccination platforms. The final mRNA molecule undergoes testing to assess its final features, such as identity, appearance, content, integrity, residual DNA, endotoxin contamination, and sterility. Finally, the ability of the mRNA to be translated into the desired protein product after delivery into target cells is checked with a potency test. Depending on the desired mRNA final characteristics, these procedures are a little adjusted to allow for nucleoside modification, new capping strategies, or adapted purification protocols [19,20]. All mRNA production protocols and instructions are following the Good Manufacturing Practice (GMP) grade, which requires manufacturers to ensure that their products are traceable, safe, pure, and effective [30]. With the coronavirus pandemic and the threat of new emerging viruses every day, we are witnessing a huge pharmaceutical industrial growth with facilities capable of producing up to 30 million doses of mRNA-based vaccines per year [31].

Types of HIV mRNA vaccines

mRNA vaccines can be further classified into self-amplifying mRNA vaccines (SAM), non-replicating mRNA vaccines, DC mRNA vaccines, mRNA vaccines for cancer. Self-amplifying mRNA vaccines create their own accessories to form dsRNA, intermediates for replication and other products [4]. They do not need any machinery to replicate their RNA after they enter the cells. SAM vaccines need a lower dose to manifest good immunologic response as compared to non-amplifying RNA vaccines. The immune response can be demonstrated from the amount of Th1-type T cells produced after the administration of the vaccine. Researchers have proven the excellent innate immune response received from patients after the administration of small doses of SAM vaccines. SAM vaccines are developed from positive-sense, single-stranded alphaviruses and are delivered as viral replicon particles (VRPs). The mRNA is administered via multiple techniques such as electroporation, cationic micelles or cationic nano-emulsion and it encodes RNA-dependent RNA polymerase along with HIV immunogen which are responsible for creating the robust immune response. Along with HIV and other viruses, SAM vaccines can be effectively used for immunity against various bacteria as well [5].

Non-amplifying mRNA vaccines comprise of antigen encoded as mRNA which is injected into the cells. It is the simplest form of the mRNA vaccine used for HIV and other pathogens. It is easy to administer; however, the dose is greater compared to the SAM vaccine due to the immunogenic response. The delivery mechanisms are like those of the SAM vaccine. With the advent of time, multiple changes have been made to non-amplifying mRNA vaccines to improve the immune response. Some of these changes comprise of using optimized codons, modifications in nucleoside and purifying the mRNA which have modified the immune response [32].

Delivery strategies of HIV mRNA vaccine

The delivery of an mRNA vaccine into the cytoplasm of antigen-presenting cells (APC) is the rate-limiting step for the vaccine activity [33]. The free mRNA HIV vaccine has stability and penetration drawbacks during transportation into APC’s cytoplasm. To improve the intake of mRNA HIV vaccine by APC, various strategies have been used.

The administration method of the vaccine plays an important role in the mRNA uptake, and it affects the overall APC response to the vaccine. Compared with intradermal and subcutaneous injections, the intranodal administration of the free mRNA HIV vaccine results in higher expression and protective antigen-specific APC responses in vitro human and in vivo mice models [34].

For many years APCs have been used for delivery of vaccines, such as DCs, which are excellent in vivo APCs to present the processed antigen to T-cells [35]. The foreign mRNA is delivered into DCs by either electroporation, using a pulse of electricity [34], or lipofection, forming a complex of special liposomes with the targeted mRNA [36] to help in penetrating the cell membrane. In an animal model, transfecting mRNA-CD34-DCs vaccine produced multiple specific T-cells against antigens, suggesting promise for new effective HIV vaccines [36].

Delivery of an mRNA vaccine can also be achieved by using nanoparticle cationic carrier formulated mRNA [37]. An experimental example of the formulated carriers is LNPs which are composed of ionizable lipids to improve the uptake of the vaccine with minimal toxic effects [38]. Administration of LNPs formulated Env-gp 160 encoded mRNA vaccine to animals produced considerable numbers of antibodies against gp 120, which is promising for a new HIV vaccination strategy. The commercially available polyethyleneimine transfection polymer has been formulated with a self-amplifying mRNA (saRNA) vaccine to induce broadly specific T-cells to produce specific antibodies for an immune response in animals. Although using naked saRNA vaccines produced good immune responses, the formulated vaccines have always shown superior effectiveness [39].

mRNA technology being used to develop HIV vaccines

Due to the recent advances in mRNA technology represented by improved delivery methods and their advantages over traditional vaccine platforms, the utilization for HIV vaccination experimentation has increased in recent years. Many preclinical studies about HIV mRNA vaccines have been published, and they are summarized in (Table 2) [35,40-45].

**Table 2:** Summary of preclinical studies of HIV mRNA vaccines. Gag: group antigen; Env: envelope; N.A.: not available.

Antigen	Delivery	mRNA type	Route	Tested Model	Immunological response
Gag	Poly (lactic acid) nanoparticles and cationic cell-penetrating peptides	Unmodified, non-amplifying	N.A.	Human monocyte-derived dendritic cells	Humoral, cellular
Env	Lipid Nanoparticles	Nucleoside-modified, non-amplifying	Intradermal	Mice, non-human primates	Humoral, cellular
Gag	Cationic nanomicelles	Unmodified, non-amplifying	Subcutaneous	Mice	Humoral
Env (trimeric gp140)	Cationic nanoemulsion	Unmodified, self-amplifying	Intramuscular	Mice, rabbits, non-human primates	Humoral, cellular
Env (trimeric gp140)	Electroporation	Unmodified, self-amplifying	Intramuscular	Mice	Humoral, cellular
Gag	mRNA transfection of dendritic cells	Unmodified, non-amplifying	N.A.	In-vitro system	Humoral, cellular

Several human clinical trials have been conducted, as illustrated in (Table 3). These trials are aimed at a therapeutic vaccine for HIV-1 using ex vivo DCs loading delivery technique. Patients that were infected with HIV-1 and undergoing highly active antiretroviral therapy were administered autologous DCs electroporated with mRNA encoding different viral antigens. This strategy was safe and effective in generating humoral and cellular immune responses, but there was no significant clinical benefit [46-52].

**Table 3:** Clinical trials for mRNA HIV vaccines. This table summarizes clinical trials for HIV mRNA vaccines registered at ClinicalTrials.gov as of June 5, 2021. TriMix:CD40L+CD70+caTLR4 RNA.

Sponsor	mRNA vaccine	Delivery	Route	Trial number	Stage	Status
Argos Therapeutics	AGS-004	Dendritic cells electroporated with autologous viral Antigen and CD40 ligand mRNAs	Intradermal	NCT02042248	Phase I	Completed
Erasmus Medical Center	iHIVARNA-01	Dendritic cells loaded in situ with viral antigen mRNA with TriMix	Intranodal	NCT02888756	Phase II	Terminated
Fundacion Clinic per a la Recerca Biomédica	iHIVARNA-01	Viral antigen mRNA with TriMix	Intranodal	NCT02413645	Phase I	Completed
Argos Therapeutics	AGS-004	Dendritic cells electroporated with autologous viral Antigen and CD40 ligand mRNAs	Intradermal	NCT01069809	Phase II	Completed with no results
Massachusetts General Hospital	PARC002	Dendritic cells loaded with viral Ag mRNA	Intradermal	NCT00833781	Phase I/II	Completed
Argos Therapeutics	AGS-004	Dendritic cells electroporated with autologous viral Antigen and CD40 ligand mRNAs	Intradermal	NCT00672191	Phase II	Completed
McGill University Health Centre	AGS-004	Autologous dendritic cells electroporated with mRNA encoding CD40 ligand and viral antigens	Intradermal	NCT00381212	Phase I/II	Completed

Advantages

Virus-caused epidemic outbreaks appear or reappear practically every year, and they are always marked by unpredictability, high morbidity, exponential expansion, and significant social and economic repercussions. The COVID-19 epidemic taught us that humankind was unprepared to deal with such perils. Scientific progress, especially in vaccine innovation and manufacturing, is needed now more than before. What is even more desirable is to work on a “vaccine on the go” approach that allows for rapid development, large-scale production, and distribution. Traditional vaccine technology, which frequently needs sophisticated and extensive research and development processes, may not be suitable for such an approach. mRNA vaccines offer various advantages over conventional vaccines, such as live-attenuated and inactivated viruses, protein subunits, and DNA vaccines [20].

Safety

Since mRNA vaccines are non-infectious and non-integrating, there is no risk of infection or insertional mutagenesis, by which an exogenous DNA sequence integrates into the genome of a host organism, causing gene dysregulation in the insertion site and possibly resulting in cellular phenotypic modification [53].

Efficacy

Nucleoside modifications have been shown to improve mRNA stability and translational capacity. LNPs can be effective carriers for delivering mRNA in vivo, resulting in rapid uptake and expression in host cells, ultimately leading to robust adaptive humoral and cellular immune responses. Furthermore, mRNA vaccination is similar to a viral infection, as the virus will be injecting its genetic material into the host cell. Both are capable of expressing antigens in situ, which induces a powerful cellular and humoral immune response. This advantage is crucial for the eradication of intracellular pathogens that require strong humoral and cytotoxic immune responses to be effective, such as HIV. Another similarity to viruses is that mRNA vaccines are capable of being recognized through specific pattern recognition receptors (PRRs), and consequently elicit innate immune responses. These are necessary for the stimulation of DCs which mature and induce subsequent adaptive immune responses [4,20,54].

Rapid Preparation and Versatility

Using completely synthetic manufacturing procedures, mRNA vaccines can be made quickly, possibly within days after acquiring gene sequence information. The platform is flexible and adaptable to various targets, making it perfect for quick responses to new infections [55]. Nucleic acid-base vaccines such as DNA and mRNA vaccines use the same manufacturing processes to synthesize different encoded antigens. The same platform can be used to produce a variety of vaccines for different pathogens, utilizing the same production and purification methods and manufacturing facilities; thus, saving enormous costs and reducing considerable time [20].

Challenges in developing HIV mRNA vaccine

Considering all the promises and advantages the mRNA vaccine has brought to the pharmaceutical industry, trials and clinical research have been multiplying in the hope of finding an effective vaccine for prophylactic and therapeutic use against HIV. Unfortunately, many of these experiments have failed.

Ex vivo loading of DCs, a delivery system typically employed in the fight against cancer, appears to be the preferable technique for HIV vaccine mRNA delivery [21]. A clinical trial was conducted in 2016 by Gandhi, et al. to immunize HIV-1 positive patients with autologous DCs transfected with an mRNA vaccine encoding Gag and Nef structural proteins. Although the sample was proved to be safe, it wasn’t eliciting sufficient and protective immune responses [50], which indicated the need to develop more immunogenic delivery systems. 

For this purpose, Zhao, et al. worked on a cationic nanoparticle carrier [PEI-stearic acid [PSA] copolymer-based] to deliver an HIV-1 Gag mRNA vaccine. The preliminary results were promising as they demonstrated enough potency, minimal toxicity, with specific antigenic immune responses [42]. Another trial conducted on macaques by Bogers, et al. worked on a self-amplifying RNA vaccine expressing HIV-1 envelope glycoprotein delivered with a cationic nanoemulsion system (CNE), the latter induced a strong cellular immune response with neutralizing antibodies [43]. 

The 2019 clinical trial on the HTI-TriMix vaccine, which is a combination of an mRNA vaccine with adjuvant molecules (Trimix), successfully completed phases I and IIa. Although it was well-tolerated, safe, and induced a potent cytotoxic T-cell response, it encountered a major throwback with the finding of an additional start codon that modified the entire antigenic protein expression [48,56].

Overcoming the challenges 

From engineering to producing and manufacturing then clinical testing, the design and production of an effective mRNA vaccine for HIV poses major challenges and obstacles putting the obligation on the scientific community for a better understanding of mRNA-based drug mechanism and delivery strategies as well as proper HIV antigen selection.

It is critical to find the right balance between the immunostimulatory activity of the mRNA vaccine and protein synthesis, as it is possible to overstimulate the innate immune system receptors which would lead to protein destruction. In this scenario, optimizing RNA synthesis manufacturing to eliminate all dsRNA has been linked to increased vaccine effectiveness [21, 55].

Because of the structure of RNA, adjuvants can be incorporated into the same molecule [57]. Finding new adjuvant compounds that can be incorporated into the RNA sequence or vaccine formulation has proved to improve vaccination efficacy, by inducing the desired effectors of an immune response [58]. The immunogen must be able to elicit a broad and powerful cytotoxic immune response, therefore it must contain epitopes that cover the most common HLA molecules while also targeting viral proteins without immunological escape [21].

The recent success of SARS-CoV-2 mRNA based-vaccines has demonstrated that LNPs are the ideal system for mRNA vaccines’ delivery [59]. Regrettably, there has been no clinical trial yet using nano-encapsulated LNP mRNA-based vaccines for HIV infection. It would be logical as a next step to test new compounds for nano-formulation of an HIV mRNA vaccine. The bad news is that there are hundreds of these molecules that may not work for any vaccine. What’s even more unfortunate, is that the best available compounds that may be ideal for an HIV mRNA vaccine are patented by pharmaceutical companies [21].

The existence of a latent reservoir is a key obstacle to an HIV operational cure. A latent reservoir is a group of CD4+ T cells that have functional HIV genes incorporated into their DNA but are not transcribed. Because viral proteins are not translated, MHC I does not provide antigen presentation, therefore CD8+ T lymphocytes are unable to recognize and kill infected cells. The CD8+ T cell response is the principal weapon used by most therapeutic HIV vaccinations to eliminate infected cells. As a result, the latent reservoir is an insurmountable barrier [60]. This is where a new approach came to the surface. A theory was stipulated to combine a therapeutic vaccine with latency reversal agents (LRA) and blocking antibodies. That’s how we can fight the virus from multiple angles. LRA are compounds that reactivate the virus, allowing the immune system to attack it. Antibodies designed against the virus, or the receptors utilized by the virus to enter cells are used to prevent the virus from spreading when the latency is broken. LRA are compounds that reactivate the virus, allowing the immune system to attack it. Antibodies designed against the HIV antigen component will prevent the virus from spreading when the latency is broken. 

Based on this principle, two research projects are making their way to make a potential HIV treatment. The first project is using a sequence coding for eight HIV fragments immunogens, termed DNA-TMEP-B, in association with immune modifying agents or LRAs, such as TLR7 agonist, IL15, and/or PD1/PDL1 pathway inhibitors [61,62]. In a phase I/IIa clinical trial, the HIVACAR project (H2020 Grant Agreement Number: 731626) will investigate a combination strategy in HIV-1 infected patients. The treatment plan will include a tailored mRNA vaccination, a CD4 blocking antibody, and an LRA. Recruitment will begin in the second half of 2021, in the prospect of finding better answers to the HIV epidemic [63]. 

## Conclusions

The goal of this review is to shed some light on the history of developments in the mRNA vaccine field as well as the studies that have been conducted to attain an mRNA vaccine for the treatment of HIV. The safety, efficacy, rapid preparation, and versatility have made mRNA vaccines a preferred platform. Understanding the history and mechanism of HIV and latest mRNA technology advances in terms of stability and delivery systems have led to great advances in the way to develop a vaccine. Delivery systems such as cationic nanoemulsions and lipid nanoparticles have shown promising results in preclinical studies. However, ex vivo loading of DCs has been the favorite technique to develop a therapeutic HIV mRNA vaccine in clinical trials. Unfortunately, these trials have not yet managed to develop a functional vaccine. The main challenges faced by these trials were high HIV antigenic variations, the presence of a latent HIV reservoir, and difficulty obtaining a broad neutralizing antibody response. Future clinical trials should focus on utilizing the concept of lipid nanoparticles as a delivery method for mRNA HIV vaccine as it already showed great results with COVID-19 vaccines. Moreover, more studies need to be conducted in order to determine the right antigen to use and overcome the obstacle of latent reservoirs are necessary for obtaining a balanced yet robust immune response.
